# The Era of Energy Drinks: Consumption Pattern, Awareness, Perception, and Their Adverse Impact on Adolescent Health in Egypt

**DOI:** 10.7759/cureus.48966

**Published:** 2023-11-17

**Authors:** Sameer Hamdy Hafez, Noha A Mohammed, Abdalla MohamedAhmed Osman, Sadeq A Alwesabi, Elsadig Eltaher Hamed Abdulrahman, Samah Ramadan Elrefaey, Mugahed Ali Alkhadher, Ateya Megahed Ibrahim, Abeer Yahia Mahdy Shalby, Mohamed ‪ Saied Harfoush

**Affiliations:** 1 Department of Community Health Nursing, Faculty of Nursing, Beni-Suef University, Beni-Suef, EGY; 2 Department of Community and Mental Health Nursing, Faculty of Nursing, Najran University, Najran, SAU; 3 Department of Community and Mental Health Nursing, College of Nursing, Najran University, Najran, SAU; 4 Department of Medical and Surgical Nursing, Faculty of Nursing, Najran University, Najran, SAU; 5 Department of Nursing, College of Applied Medical Sciences, Prince Sattam Bin Abdulaziz University, Al-Kharj, SAU; 6 Department of Family and Community Health Nursing, Faculty of Nursing, Port Said University, Damietta, EGY; 7 Department of Medical and Surgical Nursing, College of Nursing, Najran University, Najran, SAU; 8 Faculty of Nursing, Benha University, Benha, EGY; 9 Department of Community Health Nursing, Faculty of Nursing, Damanhour University, Damanhour, EGY

**Keywords:** energy drink consumption, awareness, egypt, adverse impact, adolescents

## Abstract

Background: Despite the risks associated with energy drinks (EDs), the consumption of EDs remains high, especially among adolescents, so the study aimed to assess the consumption pattern, awareness, perception of EDs, and their adverse impact on adolescent health.

Methods: A cross-sectional design was used. The study setting is Damanhur City, Beheira governorate, Egypt, with a sample size of 350 participants. A structured interview questionnaire was used to collect the data and consisted of five parts: socio-demographic data, pattern of use, negative adverse effects of EDs, knowledge, and perception.

Results: The data reveal that 38.5% of the studied sample consumed EDs, and 14.2% of them consumed more than one time per day. The main reason for consumption was feeling fatigue among 46.4%, followed by 28.7% to increase concentration. Only 36% had satisfactory awareness, and 45.7% perceived that excessive consumption has dangerous effects. Age, educational stage, gender, awareness, and perception are significant influencers on the consumption of EDs. The reported adverse impacts were polyuria among 51.4%, followed by tachycardia (40.0%) and insomnia (35%).

Conclusion: About one-third of studied adolescents consume EDs regularly and reported several adverse health effects, such as polyuria and tachycardia. The main reason for consumption was feeling fatigued among about half of the studied adolescents. Low awareness levels and negative perceptions were significantly associated with consumption.

Recommendation: Implement educational programs about EDs and their possible risks to improve the awareness level among adolescents. Further studies should be carried out across different countries.

## Introduction

Energy drinks (EDs) are beverages that typically contain large amounts of caffeine, added sugars, other additives, and legal stimulants, such as guarana, taurine, and L-carnitine. These legal stimulants can increase alertness, attention, and energy, as well as increase blood pressure, heart rate, and breathing. It was found that about 50% of the consumers are children, adolescents, and young athletes. Unfortunately, these products are sold in schools without any controls [1].

Consumption of EDs is becoming increasingly common in the world, particularly for young people. Both acceptability and availability for all age groups in a population have been enabled by the widespread advertising of these drinks, as well as their accessibility at grocery stores, convenience stores, and supermarkets. It was found that, in over 140 countries, EDs are available to be purchased and half of the consumers were children aged between 14 and 24 years [2].

The energizers in these beverages can hurtfully affect the sensory system. There were several adverse effects associated with excessive consumption, including headaches, heart palpitations, anxiety, and insomnia, with more serious side effects evident in clinical data. Consequently, researchers and health professionals have expressed concern that these beverages may cause harm when consumed in excess [3].

Change in beverage consumption intake, especially caffeinated drinks, has been reported from several parts of different Gulf countries. UAE university students reported a high prevalence of ED (85.1%) and caffeinated beverage (97%) consumption [4]. Male students from Saudi Arabia reported high use of EDs (54%). Due to an increased prevalence and popularity of caffeinated beverages among the young population, it is essential to disseminate information and knowledge regarding their use and adverse physical effects [5].

According to a previous study conducted in Egypt, about one-third of the studied university students consumed EDs regularly for several reasons as to stay awake or as refreshments, for more energy, and for better sports performance [6]. According to a recent study [7], the prevalence of ED consumption reached 74.8% among Saudi students. It is considered an emerging health risk behavior. There are also little research done on ED consumption patterns among adolescent in Egypt. In addition, there is no adequate data that report the associated factors of ED consumption. Thus, such a study is urgently needed to assess the consumption pattern, awareness, and perception of EDs and their adverse impact on adolescent health.

Research questions

What are the consumption patterns of EDs experienced by the studied adolescent?

What are the reported negative consequences associated with ED consumption among the studied adolescents?

 What are the levels of awareness among the studied adolescents about EDs?

What are the perceptions of the studied adolescents about EDs?

## Materials and methods

Research design

A cross-sectional study was adopted to carry out this study.

Study setting

The study was conducted in Damanhur City, a city in Lower Egypt, and the capital of the Beheira Governorate.

Sample size

The original sample size was 319, but the researchers increased the sample size to 350 to ensure more representation.

The sample size was estimated according to the following formula:

Sample size = (Z2) x (p) x (1-p) -------------------- m2

where:

Z = Z value (1.96 for 95% confidence level)

p = percentage of ED consumers was 29.3% [8]

m = margin of error (0.05)

Tools for data collection

One questionnaire was designed by the researcher for the collection of the required data after reviewing the following literature [1,8].

A structured questionnaire designed to collect data was composed of five parts.

Part 1: Socio-demographic characteristics and the pattern of use among the studied sample, such as age, gender, educational level, and residence.

Part 2: Pattern of use as consumption, frequency, and reasons for consumption

Part 3: The adverse impacts of ED consumption

It is an open-ended question about the most negative symptoms that occur after consumption of EDs. The participants' answers were limited to the following: thirst, tachycardia, insomnia, anxiety, increased urination, and headache.

Part 4: Knowledge questions

It is composed of 10 questions to identify the awareness of adolescents about EDs. Three multiple questions were about the following: What is the main ingredient?, How does this drink affect your body?, and Which of the following is the permitted dose of caffeine per day?. Seven are true or false questions: Are EDs alternative source of water, Are EDs associated with dental erosion, Do EDs interact with some drugs, Are EDs allowed for diabetic patients, Is consuming EDs during exercises safe?, Are EDs allowed for hypertensive patients?, Do EDs provide the body with electrolytes?, and Does the consumption EDs increase the risk of obesity?

Scoring system: correct answer (1) and incorrect answer (0). The total points are summed and converted to percentages and divided into two categories: unsatisfactory (<60%) and satisfactory if ≥60%.

Part 5: Perception questions

It is composed of three questions: Are EDs usually safe?, Does excessive consumption have dangerous effects?, and Is the consumption of EDs usually dangerous?

According to the Likert scale, every question had three choices: agree, maybe, and disagree.

Validity: The questionnaire was validated by five professors in community health nursing. The consistency by Cronbach's alpha coefficient test was 0.86.

Ethical considerations

An approval was obtained from the Research Ethics Committee of the Faculty of Nursing at Damanhur University under code number (2022-59-d). The study was conducted with careful attention to the ethical standards of research and the rights of participants. At first, verbal consent from the participants was obtained. The participants were informed about the purpose of the study, and they had the right to refuse to participate. The anonymity of the participants was maintained at all times.

Pilot study

A pilot study of 30 adolescents was conducted to evaluate the questionnaire's content and time requirements for data collection. Adolescents who participated in the pilot trial were excluded.

Inclusion criteria

All adolescents aged 15-21 years old and who accepted to participate were included in the study sample.

Field of the work

The online questionnaire was distributed from 1 January 2023 to 1 April 2023. The targeted respondents were Egyptian adolescents who lived in Behira governorate and accepted to participate in the study, with their ages ranging from 15 to 21 years old.

Statistical analysis

All available data organized into bar charts and cross-tables were used to provide an overall and coherent presentation and description of data. A packaged computer analysis program, Statistical Product and Service Solutions (SPSS, version 24.0) (IBM SPSS Statistics for Windows, Armonk, NY), was used for the statistical analysis of these data. A chi-square test was applied to determine the difference between variables. A p-value of < 0.05 was considered significant.

## Results

Table 1 shows that 37.2% of the studied sample ages ranged from 15 to 17 years old, and the rest of them ranged from 18 to 21 years old. As regards the gender and educational levels of the studied sample, 71.5% of them are males, 54.3% in university education, and 45.7% in secondary schools.

**Table 1:** Frequency distribution of the studied sample according to their socio-demographic data

Items	N	%
Age		
15-17	130	37.2
18-21	220	62.8
Mean age		
Gender		
Male	250	71.5
Female	100	28.6
Educational levels		
Secondary	160	45.7
University	190	54.3

Table 2 describes the distribution of the studied sample regarding their pattern of ED consumption. The data reveal that 38.5% of the studied sample reported that they consumed EDs before, 14.2% consumed it more than one time per day, 17.9% consumed EDs daily, 42.9% consumed EDs three to five times per week, and 25.0% of them drink EDs two to three times per week. Regarding the reasons for ED consumption as mentioned by the studied students, feeling fatigue is the main reason among 46.4% of them, followed by increased concentration (28.7%), for fun (10.0%), and for physical performance improvement (14.2%).

**Table 2:** Frequency distribution of the studied sample regarding their pattern of energy drink consumption

Items	N	%
Have you consumed energy drinks before?		
Yes	140	38.5
No	210	61.5
What is the frequency of consuming energy drinks? (140)		
1-3 times per week	35	25
4-6 times per week	60	42.9
Daily	25	17.9
More than one time per day	20	14.2
What is the main reason for energy drink consumption (140)		
Feeling fatigue	65	46.4
To increase the concentration	40	28.7
For fun	15	10.7
Improve physical performance	20	14.2

Table 3 illustrates the distribution of the studied sample regarding the negative consequences of consuming EDs. The table clarifies that the studied students suffer from negative consequences such as increased urination among 51.4%, followed by tachycardia (40.0%), insomnia (35%), headache (27.1%), and anxiety (20.7%).

**Table 3:** Frequency distribution of the studied sample regarding the negative consequences of consuming energy drinks (140) Multiple responses are considered.

Items	N	%
Tachycardia	56	40
Insomnia	49	35
Anxiety	29	20.7
Increased urination	72	51.4
Headache	38	27.1

**Figure 1:**
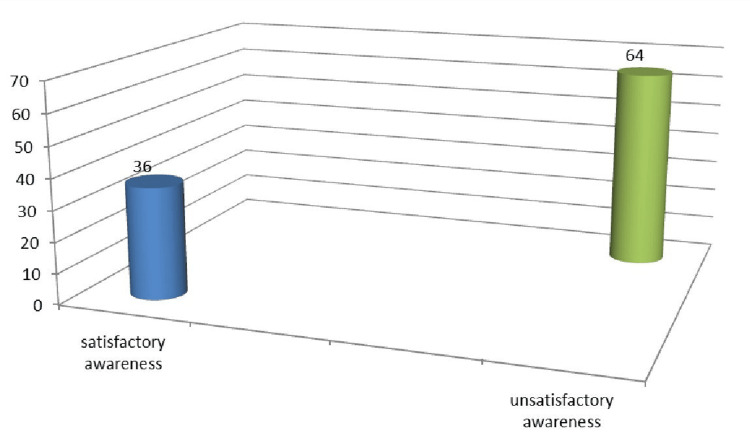
Frequency distribution of the studied sample awareness regarding the consumption of energy drinks (350)

Figure 1 illustrates that 36.0% of studied students have a satisfactory level of awareness regarding the consumption of EDs, and 64% have an unsatisfactory level of awareness.

Table 4 clarifies the distribution of the studied sample’s attitudes regarding the consumption of EDs. The data illuminate that 31.5% of studied students are convinced that EDs are usually safe, 45.7% of them reported that excessive consumption has dangerous effects, and 22.8 % believe that the consumption of energy drinks is usually dangerous. There is a highly statistically significant relation between the perceptions of the studied sample and their consumption of EDs.

**Table 4:** Frequency distribution of the studied sample perception regarding consumption of energy drinks (350)

Perception items	N	%	Consumption		X^2^	P
			Yes (140)	No (210)		
Are energy drinks usually safe?	10	31.5	67 (60.9)	43 (39.1)	29.3	0.00001
Does excessive consumption have dangerous effects?	160	45.7	50 (31.2)	110 (68.8)		
Does the consumption of energy drinks usually produce side effects?	80	22.8	23 (28.7)	57 (71.3)		

Table 5 reveals that age, gender, educational levels, and awareness level are significantly associated with the prevalence of ED consumption, where P is less than 0.05.

**Table 5:** Association of prevalence of energy drink consumption among the studied sample and their socio-demographic characteristics and levels of knowledge

Items	N	Consumption prevalence		X^2^	P
		Yes (140)	No (210)		
Age				13.5	0.0003
15-17	130	36(27.7%)	94(72.3 %)		
18-21	220	104(47.3)	116(52.7)		
Gender				25.7	0.00001
Male	250	121 (48.4)	129 (51.6)		
Female	100	19 (19)	81 (81)		
Educational stage				4.7	0.02
Secondary	160	54 (33.7)	106 (66.3)		
University	190	86 (45.2)	104 (54.8)		
Awareness level				6.7	0.009
Satisfactory	126	39	87		
Unsatisfactory	224	101	123		

## Discussion

The consumption of EDs among the Egyptian youth is still underestimated and represents a public health challenge. The current study was conducted to assess the consumption pattern, awareness, and perception of EDs and their adverse impact on adolescent health. The study found that 38.5% of the studied sample reported that they consumed EDs before, and 14.2% consumed EDs more than one time per day. The result was compatible with other studies [2,9], while it is lower than that of other studies [10]. The decreased consumption rate in the current study was because about half of the sample had a positive perception regarding the consumption of EDs.

Half of the studied adolescents reported suffering from negative consequences, such as increased urination, followed by tachycardia, insomnia, headache, and anxiety. The results were in the same line as those in the two studies [11,12], while the prevalence of negative consequences was higher than that of Alafif et al. [9]. The high rate of negative consequences among the studied sample may be due to the high consumption rate.

The current study illustrates that about one-third of studied students have a satisfactory level of awareness regarding the consumption of EDs [13,14]. The results were lower than the study conducted by Bharti et al. [15]. This difference might be because the participants were not exposed to adequate health education programs.

Regarding the perception, nearly half of the studied sample think that excessive consumption has dangerous effects, and this is considered a positive perception; these results are similar to the study conducted in Taiwan by Chang et al. [16], which is also in harmony with the results of Douglas et al. [17]; they reported that about the half had a positive attitude toward EDs. The current study has a higher percentage than the study of Ibrahim et al. [18]; they reported that only about one-fifth had a positive attitude about EDs. This may be due to the culture of the Egyptian people is that extravagance in anything is harmful to health.

The current study revealed that age, gender, educational levels, and awareness level are significantly associated with the prevalence of ED consumption. Along the same line, Puupponen et al. [19] who aimed to understand the popularity of EDs among adolescents and to identify the determinants underpinning consumption reported that gender, age, and awareness level were significant determinants for ED consumption. On the other hand, Nuss et al. [20] reported that there was no association between socio-demographic characteristics and consumption. The variation may be due to the culture in Arabic countries that are heavily affected by the socio-demographic data and levels of awareness.

Limitations of the study

Despite the contribution of this study to scientific knowledge, it cannot go without mentioning that information gathered from the respondents could be subjective because the information was self-reported. Moreover, the generalization of the results is very difficult because the sample size was small in comparing the total population and the huge variation in culture and socio-economic status.

## Conclusions

The current study revealed that about one-third consume EDs regularly and reported several adverse health effects, such as polyuria and tachycardia. The main reason for consumption was feeling fatigue among about half of the studied adolescents. The consumption of EDs was significantly associated with low awareness levels and negative perceptions regarding energy drinks.

Recommendations include implementing educational programs about EDs and their possible risks to improve the awareness level among adolescents. Further studies should be carried out across different countries.
